# Health-Related Behaviors and BMI Across Three School Years: Annual Bayesian Network Patterns During the PETICA Childhood Obesity Prevention Program

**DOI:** 10.3390/children13101298

**Published:** 2026-09-24

**Authors:** Sara Cobal Pravdić, Darija Vranešić Bender, Jasenka Gajdoš Kljusurić, Ema Kazazić, Željko Krznarić, Želimir Kurtanjek

**Affiliations:** 1 Croatian Medical Association, Šubićeva 9, 10000 Zagreb, Croatia; 2 Unit of Clinical Nutrition, Department of Internal Medicine, University Hospital Centre Zagreb, Kišpatićeva 12, 10000 Zagreb, Croatia; 3 Faculty of Food Technology and Biotechnology, University of Zagreb, Pierottijeva 6, 10000 Zagreb, Croatia; 4 School of Medicine, University of Zagreb, Šalata 2, 10000 Zagreb, Croatia

**Keywords:** prospective study, Bayesian networks, BMI, BMI-for-age percentiles, dietary habits, lifestyle behaviors, public-health program “PETICA”

## Abstract

**Highlights:**

**What are the main findings?**

- Separate annual Bayesian network models identified highly reproducible dependencies among selected health-related behaviors, particularly fruit and vegetable intake, whereas behavioral connections with BMI differed across Grades 2–4.
- The findings distinguish reproducible behavioral dependencies from year-specific associations with BMI and do not establish intervention effects.

**What are the implications of the main findings?**

- Bayesian network analysis can characterize interconnected behavioral patterns and quantify the stability of estimated network structures across repeated annual assessments.
- The observed patterns can contribute to hypotheses formation for future evaluations of multi-component school-based obesity prevention programs.

**Abstract:**

**Background/Objectives:** This study examined annual probabilistic network structures among dietary habits, health-related attitudes, lifestyle behaviors, and body mass index (BMI) during implementation of the PETICA school-based program. **Methods:** This was a three-year prospective, uncontrolled school-based intervention with repeated measurements of the same cohort in Grades 2–4. The BMI analytic samples comprised 915 pupils in Grade 2 (461 boys, 454 girls; mean age 7.9 ± 0.4), 840 in Grade 3 (429 boys, 411 girls; mean age 8.9 ± 0.5), and 684 in Grade 4 (341 boys, 343 girls; mean age 9.9 ± 0.4). BMI was recalculated from measured body mass and height and used as the continuous network outcome. BMI-for-age percentiles were retained for age- and sex-specific descriptive classification and sensitivity analysis. Separate score-based Bayesian networks were estimated for each grade. Network stability was assessed using 200 nonparametric bootstrap samples, with stable adjacency defined as a bootstrap frequency of at least 50%. **Results:** Fruit and vegetable intake formed a stable adjacency in all three grades, with bootstrap frequencies of 100.0% in Grade 2, 98.0% in Grade 3, and 99.0% in Grade 4. The sex of the pupil and computer/video-game use were stably connected in Grades 3 and 4 (100.0% in both grades). Stable connections among health-related attitudes were more prominent in later grades. In Grade 4, at-home breakfast consumption was connected with water intake (57.5%) and vegetable intake (52.0%). No behavioral adjacency with continuous BMI reached the prespecified 50% stability threshold in any grade. The largest subthreshold behavior–BMI signal was observed for at-home breakfast consumption in Grade 4 (39.0%). **Conclusions:** Selected relationships among reported health-related behaviors were reproducible across annual assessments, whereas direct behavior–BMI connections were year-specific and did not reach the stability threshold. The greater integration of attitudes and healthy habits in later grades is compatible with cumulative, age-adapted health education, but the absence of an untreated comparison group prevents attribution of these patterns to PETICA. The findings do not establish causal effects or program effectiveness, but may contribute to hypotheses generation for future evaluations of multi-component school-based obesityprevention programs.

## 1. Introduction

Childhood obesity represents one of the most significant global public health challenges of the 21st century. According to the World Health Organization, noncommunicable diseases account for approximately 90% of deaths in the European region, with obesity acting as a major contributing factor [1]. In Europe, nearly one in three children aged 7–9 years is overweight or obese, while in Croatia the prevalence reaches 36.1% among school-aged children [2]. Obesity is defined as a chronic, multifactorial disease characterized by excessive accumulation of body fat, and may impair health and increase the risk of numerous comorbidities, including cardiovascular diseases, type 2 diabetes, and psychological disorders [3,4]. Childhood obesity frequently persists into adulthood, significantly increasing long-term health risks such as an increased risk of cardiovascular events and mortality in early adulthood [5,6]. The etiology of obesity is complex and involves interactions between genetic, epigenetic, environmental, and behavioral factors. In addition, behavioral determinants that play a critical role, such as dietary patterns, physical activity, sedentary behavior and sleep, are modifiable through targeted interventions [7]. Research by Velerio et al. [8] highlights that children and adolescents with obesity have a significantly higher incidence of insulin resistance, arterial hypertension, dyslipidemia, impaired glycemia and non-alcoholic fatty liver disease compared to peers of normal body weight. In addition to metabolic complications, childhood obesity is associated with an increased risk of developing type 2 diabetes. When type 2 diabetes develops at a young age, it is associated with a higher rate of morbidity and mortality than in the later-onset form of the disease [8,9]. Overweight status and obesity in childhood and adolescence have a wide range of psychosocial burdens, including higher rates of depressive symptoms, anxiety, stigmatization, and reduced quality of life compared with children of normal weight. These psychological consequences may contribute to the vicious cycle of obesity, as emotional distress leads to emotional overeating, reduced physical activity and poorer adherence to intervention programs [6]. A review focused on long-term consequences of childhood obesity shows that it also increases the risk for various chronic diseases, including cardiovascular disease, certain autoimmune diseases, and premature mortality, with mortality rates up to age 30 being several times higher than in a population of normal weight [9]. A cohort study [10] showed that a good response to treatment of childhood obesity significantly reduces the risk of developing type 2 diabetes, dyslipidemia, and hypertension, as well as premature mortality, in youth. Complete remission of childhood obesity is associated with the greatest reduction in cardiometabolic risk, highlighting the need for early and intensive interventions [10,11,12]. School-based interventions are recognized as one of the most effective strategies for childhood obesity prevention. Schools provide a structured environment, ensure access to large populations over a longer period of time, and allow for continuous monitoring and implementation of health-promoting activities [5,13,14,15]. Furthermore, involving parents enhances intervention effectiveness, as parental behaviors strongly influence children’s lifestyle habits [5,16,17]. Despite extensive intervention efforts, the multifaceted etiology of obesity calls for analytical approaches capable of modeling the complex dependencies among its behavioral, environmental, and biological determinants [18]. Traditional statistical methods often fail to model such complexity adequately. In this context, Bayesian networks (BNs) offer a flexible framework for representing conditional dependencies between variables [19]. BNs enable the modeling of complex systems and the identification of variables that occupy prominent positions within an estimated network, without establishing cause-and-effect relationships themselves.

Understanding how health-related behaviors are interconnected can inform the design of school-based prevention programs. The “PETICA—Play for Health” model is a multicomponent educational program addressing dietary habits, physical activity, sedentary behavior, sleep, and parental engagement [20,21,22]. The present study aimed to estimate and compare annual Bayesian network structures of health-related behaviors and continuous BMI in the same cohort from Grade 2 to Grade 4 during program implementation. Observed changes were not interpreted as intervention effects because no untreated comparison group was available.

## 2. Materials and Methods

### 2.1. Study Design and Participants

This was a three-year prospective, uncontrolled school-based intervention with repeated measurements of the same cohort. Three educational cycles were implemented in 16 primary schools in Zagreb, Croatia, from Grade 2 to Grade 4. At baseline, 915 pupils participated. Because schools were not selected using probability sampling, the sample is described as a large multisite school sample rather than as representative of all children in the target population. The basic data of participants is shown in Table 1.

An a priori calculation for the original behavioral intervention objective indicated that 460 participants were required at the final assessment (effect size d = 0.5, alpha = 0.05, power = 0.95) [23,24]. Assuming 26% cumulative attrition over the follow-up [25], the corresponding baseline target was 460/(1 − 0.26) = 622 pupils. The actual baseline enrolment of 915 exceeded this target, and 684 pupils contributed BMI data in G4. This calculation was not designed to determine power for Bayesian-network structure learning, for which no single closed-form sample-size formula applies for the present mixed-node model. Uncertainty in the network analysis was therefore quantified using 200 nonparametric bootstrap samples and prespecified edge-stability thresholds.

**Table 1:** Basic data about participants of the PETICA program.

Year (Grade)	Sex	N	Body Mass (kg)	Body Height (cm)
Year 1 (2nd grade, 7.5–8.5 y old)	Boys	461	32.5 ± 6.4	136 ± 5.8
	Girls	454	32.2 ± 6.8	135.1 ± 6.1
Year 2 (3rd grade, 8.5–9.5 y old)	Boys	429	36.7 ± 7.6	142.0 ± 6.0
	Girls	411	37.1 ± 8.4	141.5 ± 6.6
Year 3 (4th grade, 9.5–10.5 y old)	Boys	341	41.5 ± 9.0	147.3 ± 6.6
	Girls	343	40.9 ± 10	147.3 ± 7.4

### 2.2. Intervention Model

The “PETICA” model is based on the Ensemble Prévenons l’Obésité Des Enfants (EPODE) methodology [26]. The Croatian model is a multi-level, school-based program that was designed by an interdisciplinary team of experts. The focal points of the intervention are the pupils and their guardians, but it also includes the local community [19]. Community-engaged approaches have also been investigated as a strategy for improving weight-related and behavioral outcomes in childhood obesity prevention [26,27].

The “PETICA” program includes (i) educational lectures and workshops; (ii) promotion of healthy dietary habits; (iii) encouragement of physical activity; (iv) reduction of sedentary behavior; and (v) active involvement of parents.

The intervention targeted key behavioral determinants linked to obesity risk via education during the school year. The educational materials used by the educators (teachers) for the lectures and workshops implemented in the school curriculum were approved by the Croatian Education and Teacher Training Agency. The educational materials used by the “PETICA” model are based on nutritional and healthy lifestyle guidelines for primary-school children [28], and emphasize the importance of a balanced, nutritious diet, which entails consuming fruits and vegetables, whole grains (as an important source of fiber), and low-fat milk and dairy products and limiting ultra-processed foods, fast food, industrially produced sauces, sweetened carbonated drinks, and snacks/sweets that contain added sugar and salt [28,29]. They are also focused on frequent physical activity and limited sedentary behavior and screen time during the day [28,29]. Lastly, the intervention is implemented among pupils by focusing on their attitudes, knowledge and habits regarding key topics and recommendations: (i) regular breakfast consumption; (ii) fruit and vegetable intake (≥five portions/daily); (iii) physical activity (≥60 min/daily); (iv) sleep duration (9–11 h/nightly); (v) water intake (five to seven cups/daily) and (vi) screen time (<2 h/daily).

The program is designed for three consecutive school years (Grades 2, 3 and 4), with grade-specific educational content and annual measurement. The study design and analytical framework are presented in Figure 1.

### 2.3. Data Collection

Data collection was conducted as a part of a three-year prospective, uncontrolled school-based intervention with repeated annual measurements (2015/2016–2017/2018). Data were gathered within the school curriculum using two primary methods: (i) self-administered questionnaires and (ii) anthropometric measurements.

#### 2.3.1. Questionnaire

The structured, self-administered questionnaire contained 11 harmonized items used for all three assessments. General behavior items comprised television viewing, computer/video-game use, and nocturnal sleep duration. Attitude items assessed the perceived importance of healthy eating and physical activity and whether proper nutrition and physical activity were related to health. Habit items assessed at-home breakfast consumption, water intake, fruit intake, vegetable intake, and participation in sport or physical activity. Exact item wordings and response options are provided in the Supplementary Material.

Before the main assessment, two rounds of pilot testing were conducted with pupils in Grade 2 to refine wording and response options for age appropriateness, clarity, and comprehension. Because no psychometric reliability or construct-validity coefficients were obtained, this procedure is described as pilot testing rather than formal questionnaire validation.

The questionnaire was administered to the same cohort of pupils at three annual assessment points: in Grades 2, 3, and 4 (G2, G3, and G4). Data collection was carried out during school hours under teacher supervision to ensure standardized administration.

The study was conducted in accordance with ethical standards for research involving human participants. Ethical approval was obtained from the relevant institutional ethics committee, and permission to conduct the study was granted by school authorities. Participation was voluntary, and informed consent was obtained from parents or legal guardians prior to data collection.

#### 2.3.2. Anthropometric Measurements

Body mass (BM) and body height (BH) were measured once per school year by trained medical personnel. Measurements were recorded to the nearest 0.1 kg and 0.1 cm using a calibrated Beurer PS06 scale (Beurer, Ulm, Germany) and a SECA 217 stadiometer (Seca, Hamburg, Germany). Pupils were measured barefoot and in light clothing. BMI was calculated as body mass in kilograms divided by height in meters squared (kg/m^2^). Age- and sex-specific BMI-for-age percentiles based on Croatian reference values [30] were used to describe nutritional status: overweight was defined as the 85th to <95th percentile and obesity as >=95th percentile. Continuous BMI was retained for network modelling because conventional percentile values compress variation at the upper end of the distribution; BMI-for-age percentiles were retained for age- and sex-specific descriptive classification. For analysis, BMI was recalculated for every record from measured body mass and height; this corrected seven G2 imported values in which the decimal separator had been lost.

### 2.4. Data Analysis

The collected data were analyzed using univariate, bivariate, and multivariate statistical methods, depending on the type of variables.

#### 2.4.1. Questionnaire Analysis

Questionnaire variables were analyzed as categorical variables, using their original ordered response codes. Television viewing was coded as 1 = ‘no television’, 2 = <=1 h/day, 3 = 2–4 h/day, 4 = >4 h/day, and 5 = ‘I do not know’. Computer/video-game use was coded as 1 = <=1 h/day, 2 = 2–4 h/day, 3 = >4 h/day, and 4 = ‘I do not know’. Sleep was coded as 1 = <6 h/night, 2 = 6–8 h, 3 = 9–11 h, and 4 = ‘I do not know’. Each attitude item was coded as 1 = ‘yes’, 2 = ‘yes, sometimes’, 3 = ‘no’, and 4 = ‘I do not know’. At-home breakfast consumption was coded as 1 = ‘every morning’, 2 = 2–3 times/week, 3 = ‘no meal before school’, and 4 = ‘I do not know’. Water intake was coded as 1 = ‘no water’, 2 = 1–2 glasses/day, 3 = 3–4 glasses/day, and 4= >=5 glasses/day. Fruit and vegetable intake were each coded as 1 = none on the previous day, 2 = once, 3 = twice, and 4 = three or more times. Sport/physical activity was coded as 1 = at least 60 min every day, 2 = 60 min 2–3 times/week, 3 = ‘rarely active’, and 4 = ‘I do not know’. The source code 999 denoted a missing response and was not treated as a substantive category. In a prespecified sensitivity analysis requested during peer review, sleep was recoded as <9 h, 9–11 h, and ‘I do not know’, with missing responses retained separately. Sex differences in Grade 2 were assessed using the chi-square test of independence. Changes across grades were assessed using the Stuart–Maxwell test only among pupils with linkable paired observations for the relevant comparison. Statistical significance was set at *p* < 0.05.

#### 2.4.2. Anthropometry

Anthropometric data of pupils were analyzed using descriptive statistics and graphical methods. Data distribution was assessed for normality, and results were presented accordingly. Continuous anthropometric variables were visualized using box-and-whisker plots to illustrate the median, interquartile range, variability, and potential outliers across measurements. In addition, the assumption of normality was evaluated to determine the appropriate descriptive and inferential statistical approach. Probability density distributions were estimated using kernel density estimation (KDE) and Orange Data Mining was used for data visualization. This approach allowed a comprehensive overview of the distributional characteristics of the anthropometric measures.

#### 2.4.3. Bayesian Network Models

Bayesian network models were used to estimate conditional dependencies among sex, reported behaviors, health-related attitudes, and continuous BMI. A separate directed acyclic graph (DAG) was learned for each grade; consequently, these analyses represent three separate annual networks rather than a single longitudinal network linking earlier exposures to later outcomes. Network structure was estimated using greedy score-based hill climbing with the Bayesian information criterion (BIC). Categorical nodes were scored using a discrete multinomial local model, and the continuous BMI node was scored using a conditional linear-Gaussian local model with dummy-coded categorical parents.

Sex was constrained as an exogenous node, and BMI was constrained as an outcome node with no outgoing edges. No other edges were required a priori. To reduce overfitting, the maximum number of parents per node was limited to three. Missing questionnaire responses, originally coded as 999, were treated as a dedicated missing category; observations without BMI were excluded from the corresponding annual BMI network. Network stability was assessed using 200 nonparametric bootstrap samples. For each pair of nodes, adjacency frequency was calculated as the proportion of bootstrap networks containing an edge between them, irrespective of direction. Adjacencies present in at least 50% of bootstrap networks were considered stable. Direction was only summarized conditional on the presence of an adjacency and was not interpreted causally.

The joint probability distribution factorizes according to the network structure as

*P*(*X1*, *X2*, …, *Xn*) = ∏ *P*(*XI* | *Parents*(*Xi*))

where each variable is conditionally independent of its non-descendants given its parents in the DAG.

By integrating probabilistic reasoning with graphical structures, Bayesian networks allow inferences about conditional dependencies among measured variables. In public health research they can identify variables and connections that recur within a multivariate system and generate hypotheses for subsequent controlled evaluation. In the present study, the models were used to describe annual dependency patterns and they bootstrap reproducibility without additional assumptions and experimental evidence; they do not identify mechanisms of action or causes of obesity risk.

The variables included in the network modeling were as follows:

- Exogenous factor: sex (x1);
- General habits: TV time (x2), video game/computer time (x3), and sleep duration (x4);
- Attitudes: perceived importance of healthy eating (x5), perceived importance of physical activity (x6), and perceived relationship of proper nutrition and physical activity with health (x7);
- Habits: breakfast consumption frequency at home (x10), water intake (x11), fruit intake (x12), vegetable intake (x13), and participation in sport or physical activity (x14)
- Outcome variable: continuous BMI in kg/m^2^ (x17). BMI-for-age percentile was used for descriptive nutritional-status classification and in a sensitivity analysis.

The Bayesian network analyses were performed in the BioCompute Notebook (BNs) environment, using Python 3.10. The computational workflow used numpy 1.26, pandas 2.1, etwork 3.2, scipy 1.11, matplotlib 3.8, and seaborn 0.13. The specified Python version and package set support reproducibility of the network computations and visualizations. The networks were used to describe conditional dependencies and their reproducibility across grades; they were not used to claim causal effects of the PETICA program.

## 3. Results

Analyses were conducted at three annual assessments. The baseline BMI network included 915 pupils (461 boys and 454 girls). BMI was available for 840 pupils in Grade 3 (429 boys and 411 girls) and 684 pupils in Grade 4 (341 boys and 343 girls), corresponding to a 25.2% reduction in annual BMI availability from G2 to G4. This was similar to the approximately 26% attrition reported in comparable longitudinal research [17]. Deterministic linkage identified 733 G2–G3 pairs and 593 G2–G4 pairs. The lower linked count also reflects inconsistent identifiers across annual files and should not be interpreted solely as biological or program-related dropout. Baseline characteristics were compared between pupils deterministically linked from G2 to G4 and pupils not linked to G4. Comparisons included BMI, BMI-for-age percentile, sex, and the 11 harmonized questionnaire variables. After Benjamini–Hochberg correction for 14 comparisons, no baseline difference remained statistically significant (all q ≥ 0.405). Questionnaire response frequencies and the corresponding sex- and grade-comparison tests are reported in Supplementary Table S1.

### 3.1. Changes in Health-Related Attitudes and Behaviors Across Grades

Overall, awareness of healthy lifestyle principles was consistently high across all grades. More than 96% of pupils, regardless of gender, reported that healthy eating and physical activity are important and related to health, with no significant changes over time. Sedentary behaviors showed no consistent improvement. Time spent watching television remained relatively stable across grades, with a substantial proportion of pupils continuing to report prolonged viewing. In contrast, computer and video game use varied significantly over time (*p* < 0.05), with a temporary reduction in daily usage in the third grade—particularly among girls—followed by a partial increase in the fourth grade. Boys consistently reported higher levels of prolonged screen use, compared to girls (*p* < 0.001). Sleep duration remained stable throughout the study period, with the majority of pupils reporting 9–11 h of sleep per night and there were no significant differences across grades or between genders.

At-home breakfast consumption increased from approximately 75% reporting daily breakfast in Grade 2 to more than 96% in Grade 3 and remained above 90% in Grade 4 (*p* < 0.001). Water intake showed no statistically significant change. Fruit and vegetable intake declined in Grade 3 and increased again in Grade 4 (*p* < 0.001). Reported daily physical activity decreased between Grades 2 and 3 (*p* < 0.01) and partially increased in Grade 4. These are observed changes during the program period and they cannot be attributed to the intervention in the absence of an untreated comparison group.

### 3.2. Anthropometric Outcomes

Changes in anthropometric parameters (body height, body mass, and body mass index) were analyzed separately by sex and the grade level of the child. The box-and-whisker diagrams (Figure 2) and distributions of these variables are presented using density plots (Figure 3), providing insight into central tendency, variability, and the presence of outliers across measurement points.

Nutritional status was assessed using age- and sex-specific BMI-for-age percentiles based on national Croatian growth reference curves developed using the Lambda–Mu–Sigma (LMS) method. According with standard percentile cut-offs, participants were classified as overweight (≥85th percentile) or obese (≥95th percentile). Descriptive results are summarized in the table below, presenting mean values of anthropometric parameters alongside the proportions of participants classified as overweight or obese (Table 2). In addition, the distribution of BMI-for-age percentiles is illustrated graphically, allowing for a more detailed assessment of the sample structure.

Analysis of higher-order distribution moments indicated mild platykurtosis and positive skewness in BMI-for-age percentiles (Table 3). A slight increase in kurtosis was observed with advancing grade level, suggesting a gradual flattening of the distribution, independent of sex.

Mean BMI-for-age percentiles were close to the center of the reference distribution, while the large standard deviations indicated substantial between-pupil variability. Positive skewness reflected a longer upper tail. Conventional percentiles also compress differences at the extreme upper end of BMI; consequently, continuous BMI was used for the primary network analysis, while BMI-for-age percentile was retained for descriptive classification and sensitivity analysis.

### 3.3. Bayesian Network Analysis

The bootstrap analysis identified two stable adjacencies in Grade 2, three in Grade 3, and seven in Grade 4 (Table 4; Figure 4). Fruit and vegetable intake formed the only stable adjacency reproduced in all three grades, with bootstrap frequencies of 100.0%, 98.0%, and 99.0%, respectively. Television viewing and computer/video-game use were strongly connected in Grade 2 (99.0%). The sex of the pupil and computer/video-game use were connected in Grades 3 and 4 (100.0% in both years). Connections among health-related attitudes were observed in Grades 3 and 4, and Grade 4 additionally showed stable adjacencies between breakfast at home and water intake (57.5%) and between breakfast at home and vegetable intake (52.0%).

No behavioral adjacency with BMI reached the 50% stability threshold in any grade. The largest subthreshold signal was between at-home breakfast consumption and BMI in Grade 4 (39.0%); the largest G2 signal was vegetable intake (7.0%); and the largest G3 signal was sex (12.0%). In the sensitivity analysis using continuous BMI-for-age percentile, no behavioral connection with the outcome was stable across all three grades. Recoding sleep as <9 h, 9–11 h, and ‘I do not know’ also produced no stable sleep–BMI adjacency (0.5% in G2, 2.5% in G3, and 3.5% in G4). Pairwise similarity of the sets of stable BMI-network adjacencies was modest, indicating that the full annual network structure was not replicated unchanged over time.

## 4. Discussion

The principal finding was a distinction between reproducible dependencies among selected reported behaviors and year-specific connections with BMI. Fruit and vegetable intake remained strongly adjacent at all three assessments, suggesting a persistent clustering of these dietary behaviors. The sex of the pupil was strongly connected with computer/video-game use in Grades 3 and 4, consistent with the observed sex differences in screen-related behavior. Connections among health-related attitudes became more prominent in the later grades.

No behavioral connection with BMI reached the prespecified stability threshold in any grade. At-home breakfast consumption showed the largest subthreshold connection with BMI in Grade 4 but not in the earlier grades. These findings do not support the original interpretation that breakfast, sleep, screen time, and physical activity were stable determinants of nutritional status across all three years [12,13,14,15]. Instead, they indicate that behavior–BMI connections were sensitive to assessment year and outcome definition.

The annual networks showed an increasing number of stable connections among health-related attitudes in the later grades. In Grade 4, at-home breakfast consumption was also connected with water and vegetable intake, suggesting that health-related behaviors may increasingly occur as coordinated patterns rather than as isolated practices. These findings were observed during the period in which pupils received repeated, age-adapted education through the PETICA program [19,22]. Pupils completed the questionnaire at the beginning of each school year and subsequently participated in educational activities addressing healthy nutrition and physical activity, with new content introduced at each grade level. The observed network patterns are therefore compatible with the possibility that cumulative educational exposure contributed to a more integrated understanding and organization of healthy behaviors. However, because the study did not include an untreated comparison group [13,14,15,16,17], these changes cannot be attributed specifically to PETICA and may also reflect maturation, family and school influences, secular trends, repeated questionnaire administration, or changes in the measurement instrument [16,17,18].

In contrast, direct connections between individual behaviors and BMI did not reach the prespecified stability threshold. Anthropometric development during middle childhood is dynamic and reflects changes in height, body mass, body composition, growth velocity, biological maturation, and sex-related developmental trajectories [3,21]. Consequently, a behavioral change or an improvement in health-related knowledge may not be accompanied by a concurrent or readily detectable change in BMI. BMI-for-age percentiles account for age and sex in the clinical description of nutritional status [30], but their bounded scale may compress differences at the extreme upper end of the BMI distribution. Continuous BMI was therefore used as the primary network outcome, while BMI-for-age percentiles were retained for age- and sex-specific descriptive classification and sensitivity analysis. Because raw BMI is not age-standardized, estimating each grade separately and including sex in the network reduced but did not eliminate developmental comparability concerns. This distinction is relevant in the broader context of the persistently high global burden of childhood overweight status and obesity [31,32], as prevention programs may influence health-related behaviors even when corresponding changes in BMI are not immediately detectable. Several limitations should be considered. The study was uncontrolled and school selection was not probability based. Reasons for non-participation at an individual assessment were not documented and may have included absence on the measurement day, relocation, or school transfer. Behaviors were self-reported, and ‘I do not know’ responses were common for some items. Exact age in months was unavailable in the analytic files, attrition may have introduced selection bias, and changes to some questionnaire items required restriction to the 11 harmonized questions available in all three grades. Bayesian-network direction is not uniquely identified for many observational structures, and unmeasured socioeconomic, familial, genetic, and neighborhood factors may confound the observed dependencies. Bootstrap stability quantifies sampling reproducibility but does not establish causality or external validity.

## 5. Conclusions

Separate annual Bayesian networks identified a reproducible connection between fruit and vegetable intake and additional grade-specific dependencies among screen behaviors, health-related attitudes, and breakfast-related habits. The findings suggest that repeated, age-adapted health education may be more readily reflected in the organization of attitudes and behavioral patterns than in stable direct connections with BMI. Although this interpretation is compatible with the educational structure of the PETICA program, the absence of an untreated comparison group prevents attribution of the observed annual differences to the program itself. No behavioral connection with continuous BMI was stable in any grade. The findings support the use of network analysis for identifying clusters of behaviors and generating intervention hypotheses, but they do not demonstrate causal effects or establish the effectiveness of PETICA.

Future controlled studies should test whether the observed network patterns differ from natural developmental changes and should use prespecified, age-appropriate adiposity metrics and external validation of network stability.

## Figures and Tables

**Figure 1:**
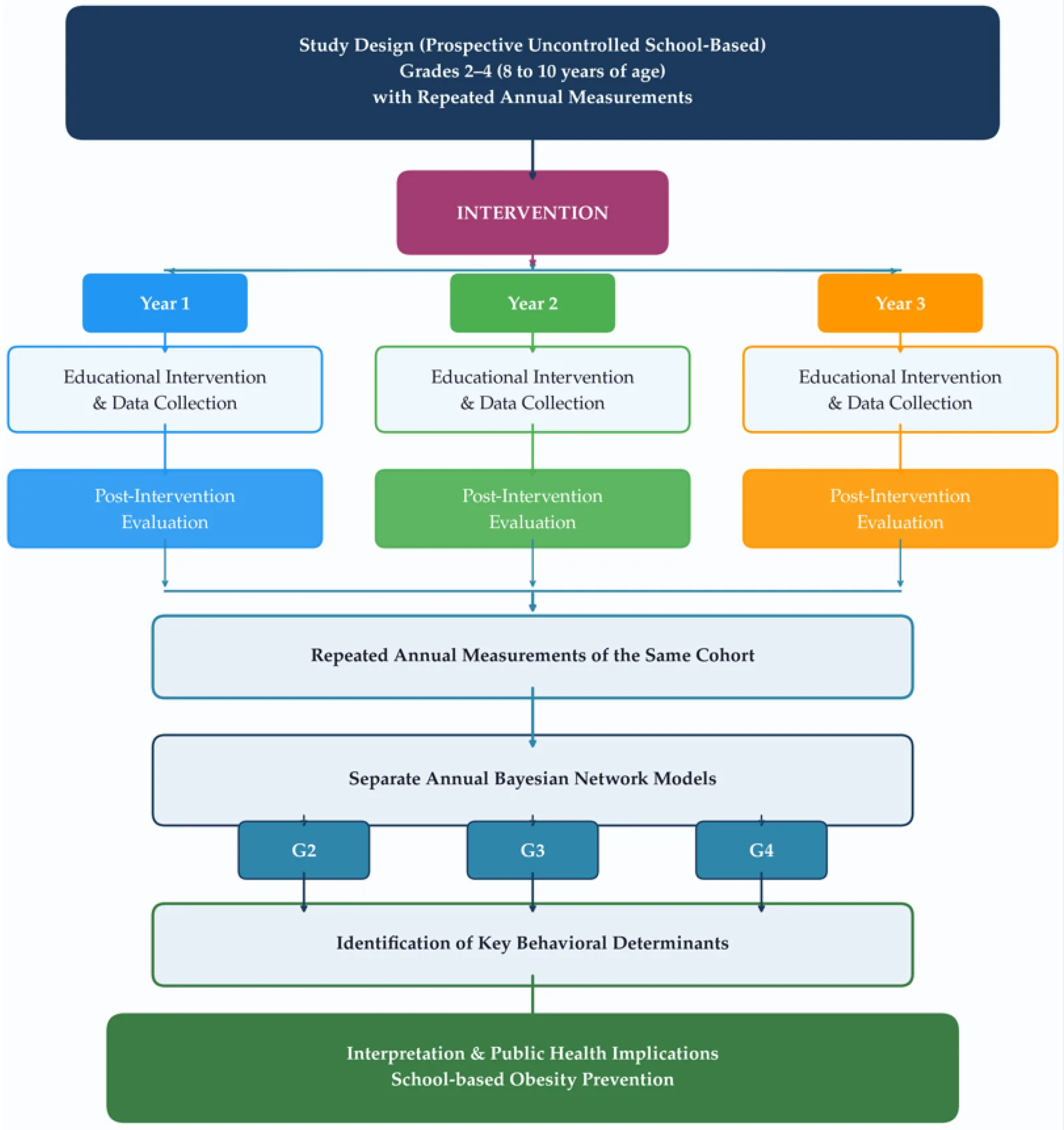
Study design and analytical framework of the PETICA three-year school-based intervention, utilizing repeated annual Bayesian network models.

**Figure 2:**
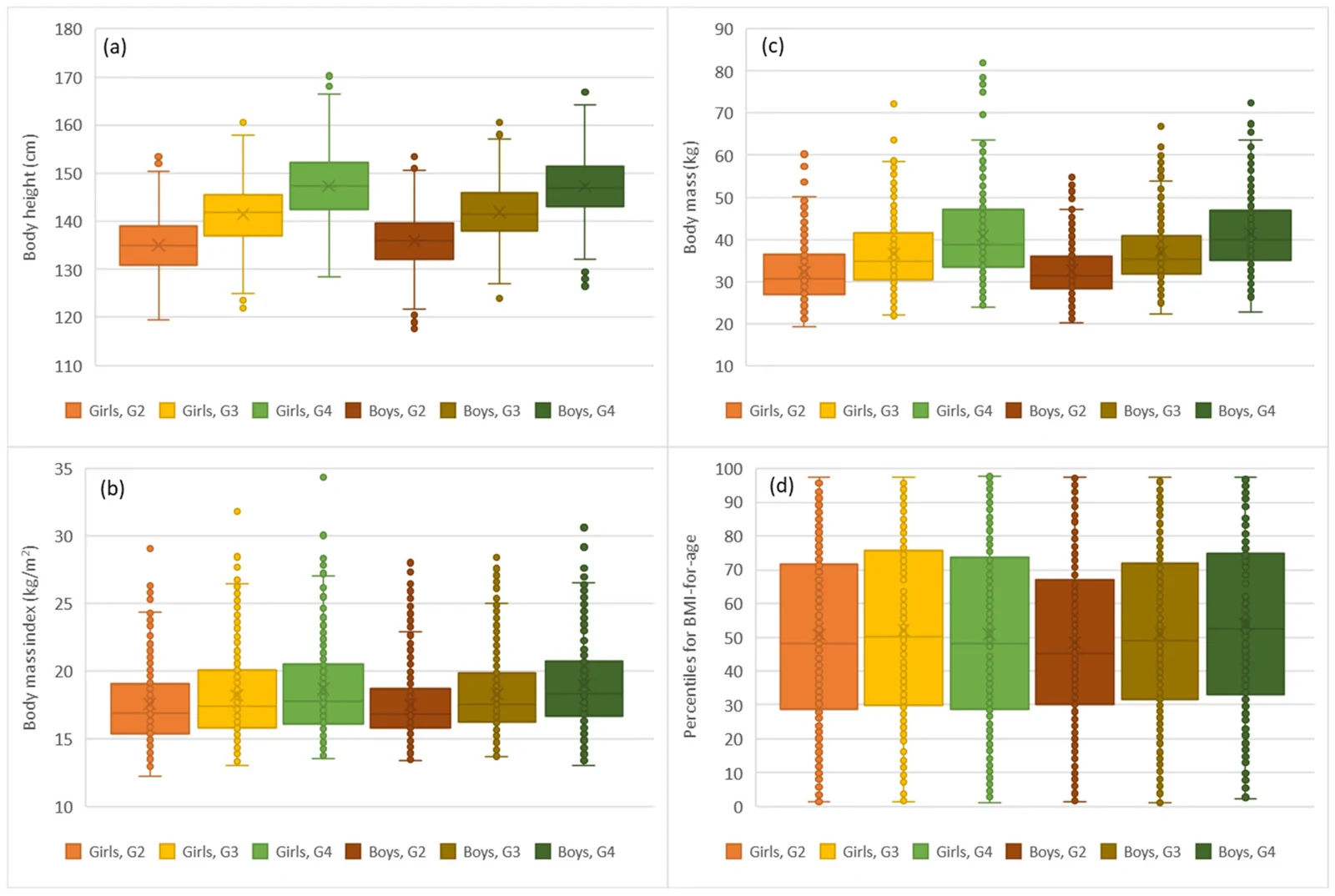
Distribution of body height (**a**), body mass (**b**), body mass index (**c**) and percentiles of BMI-for-age (**d**) in children across Grade 2 (G2), Grade 3 (G3), and Grade 4 (G4), presented as box-and-whisker plots stratified by sex.

**Figure 3:**
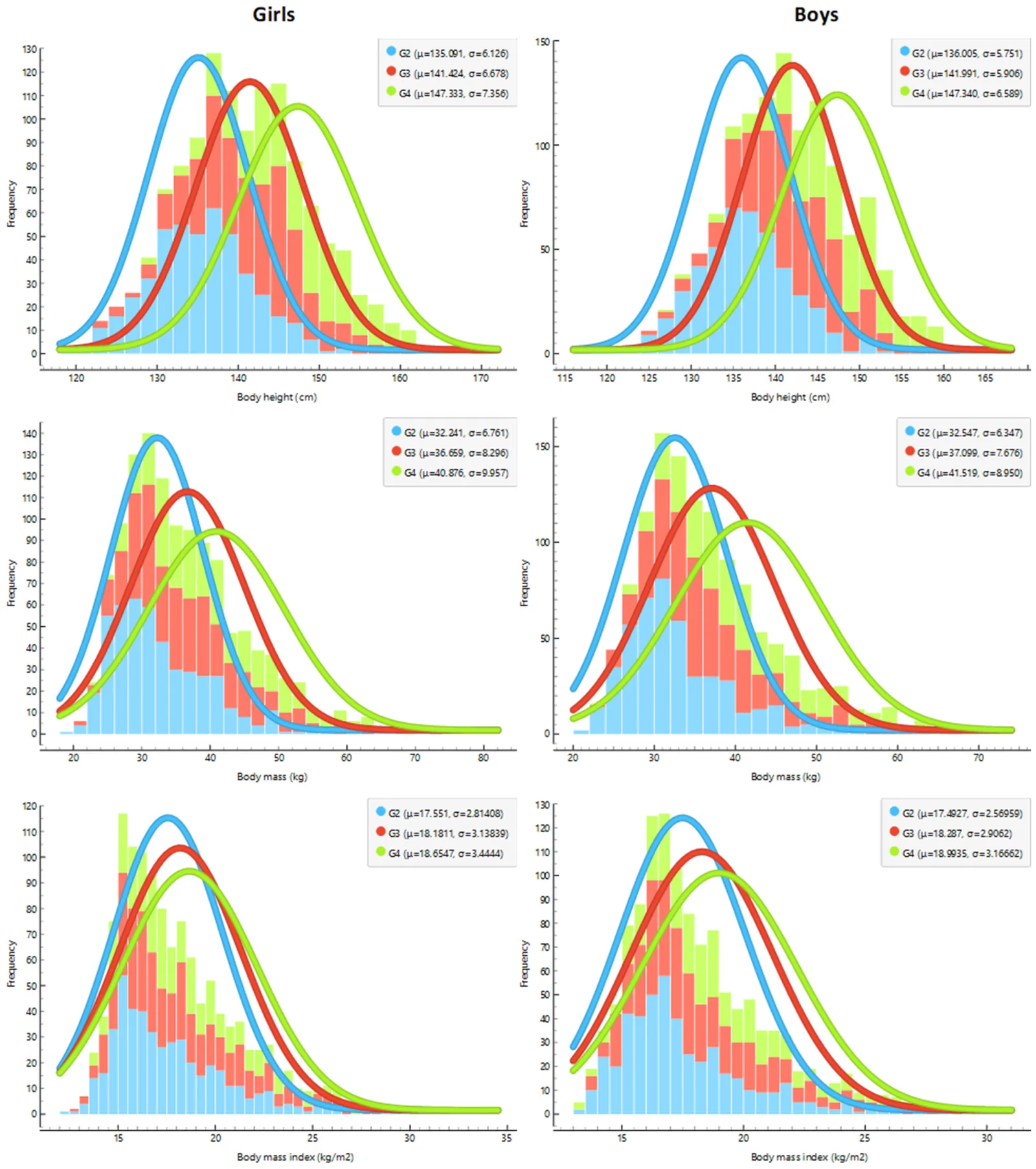
Distributions of anthropometric parameters of pupils over time (G2, G3, and G4), presented with density plots.

**Figure 4:**
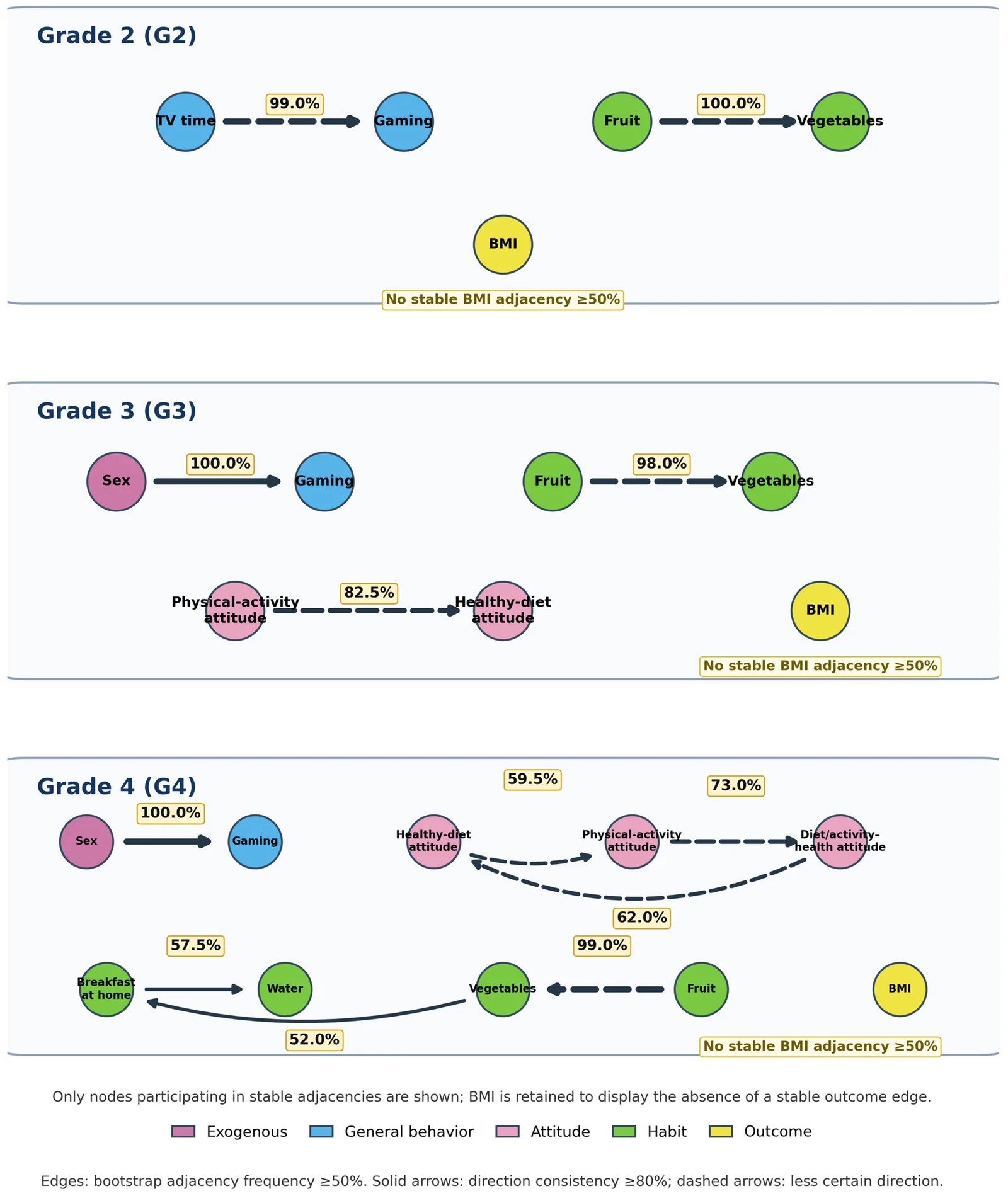
Stable annual Bayesian-network adjacencies with continuous BMI as the outcome in Grades 2–4. Grades are displayed in separate panels. To reduce visual clutter, only nodes participating in adjacencies occurring in at least 50% of 200 bootstrap networks are shown; BMI is retained in each panel to indicate that no stable outcome adjacency was identified. Edge labels denote bootstrap adjacency frequencies. Solid arrows indicate a majority direction observed in at least 80% of bootstrap networks containing the adjacency; dashed arrows indicate a direction that is less certain. Edge direction is descriptive and should not be interpreted causally. Node colors identify exogenous factors, general behaviors, attitudes, habits, and the BMI outcome.

**Table 2:** Mean BMI-for-age percentile (±SD) and prevalence of overweight status and obesity by grade and sex.

Year of the Study (Grade)	Sex	N	Percentiles (BMI-for Age)		
			$\bar{x}$ ± SD	Overweight: 85th–95th (%)	Obese: >95th (%)
Year 1 (Grade 2, G2)	Boys	461	48.3 ± 25.3	7.5	3.7
	Girls	454	50.7 ± 26.1	8.8	4.6
Year 2 (Grade 3, G3)	Boys	429	49.3 ± 25.8	6.3	4.4
	Girls	411	54.1 ± 26.6	10.2	7.1
Year 3 (Grade 4, G4)	Boys	341	53.7 ± 25.7	10.0	5.6
	Girls	343	50.9 ± 27.3	8.7	6.7

**Table 3:** Central moments presenting skewness (α_3_) and kurtosis (α_4_) of the distribution of percentiles of BMI-for-age of Croatian pupils.

Moments	Values of Central Moments of Skewness (α_3_) * and Kurtosis (α_4_) ^#^					
	Boys			Girls		
	G2	G3	G4	G2	G3	G4
α_3_	0.178	0.104	0.018	0.114	0.098	0.098
α_4_	2.189	2.064	2.003	1.979	1.848	1.891

* |α_3_| in the range 0–0.25: negligible asymmetry; 0.25–0.5: weak asymmetry; 0.5–0.75: moderate asymmetry and >0.75: strong asymmetry; α_3_ > 0 ⟹ positive asymmetry (asymmetry to the right); α_3_ = 0 ⟹ no asymmetry (asymmetry to the right); α_3_ < 0 ⟹ negative asymmetry (asymmetry to the left); ^#^ α_4_ > 3 ⟹ elongated; α_4_ = 3 ⟹ normal distribution; α_4_ < 3 ⟹ flattened. G2: Grade 2 pupil; G3: Grade 3 pupil; G4: Grade 4 pupil.

**Table 4:** Stable annual adjacencies (bootstrap frequency >=50%) in Bayesian networks with continuous BMI as the outcome.

Grade	Stable Adjacency	Frequency	Majority Direction (Consistency)
G2	Fruit—Vegetables	100.0%	Fruit → Vegetables (64.5%)
G2	TV time—Gaming	99.0%	TV time → Gaming (54.5%)
G3	Sex—Gaming	100.0%	Sex → Gaming (100.0%)
G3	Fruit—Vegetables	98.0%	Fruit → Vegetables (70.4%)
G3	Healthy-diet attitude—Physical-activity attitude	82.5%	Physical-activity attitude → Healthy-diet attitude (73.9%)
G4	Sex—Gaming	100.0%	Sex → Gaming (100.0%)
G4	Fruit—Vegetables	99.0%	Fruit → Vegetables (69.2%)
G4	Physical-activity attitude—Diet/activity– health attitude	73.0%	Physical-activity attitude → Diet/activity– health attitude (55.5%)
G4	Healthy-diet attitude—Diet/activity– health attitude	62.0%	Diet/activity– health attitude → Healthy-diet attitude (55.6%)
G4	Healthy-diet attitude—Physical-activity attitude	59.5%	Healthy-diet attitude → Physical-activity attitude (58.0%)
G4	At-home breakfast consumption—Water	57.5%	At-home breakfast consumption → Water (80.0%)
G4	At-home breakfast consumption—Vegetables	52.0%	Vegetables → At-home breakfast consumption (85.6%)

## Data Availability

The data presented in this study are available on request from the corresponding author, due to restrictions associated with privacy.

## Supplementary Materials

The following supporting information can be downloaded at: https://www.mdpi.com/article/10.3390/children13101298/s1, Figure S1: Pilot-tested questionnaire for pupils in Grade 2, Grade 3, and Grade 4; Table S1: Frequencies of responses on habits and attitudes among pupils across Grades 2–4 (total sample and by sex), with tests of statistical significance.

## Abbreviations

The following abbreviations are used in this manuscript:

BNs	Bayesian networks
EPODE	Ensemble Prévenons l’Obésité Des Enfants
BM	Body mass
BH	Body height
BMI-for-age	Body mass index-for-age
KDE	Kernel density estimation
DAG	Directed acyclic graph
LMS	Lambda–Mu–Sigma
