# Supplementary material for: Health-Related Behaviors and BMI Across Three School Years: Annual Bayesian Network Patterns During the PETICA Childhood Obesity Prevention Program
Source: Children (Basel). 2026 Sep 24;13(10):1298. doi: 10.3390/children13101298 (PMC13703612; doi:10.3390/children13101298)
Supplement: Supplementary file 1 [file children-13-01298-s001.zip › Figure S1_Pilot tested Questionairre_PETICA.pdf]

Questionnaire for pupils

1. **How much time do you spend watching TV per day?**
a) I don't watch TV
b) 1 hour or less
c) 2 to 4 hours
d) More than 4 hours
e) I don't know

2. **How often do you play on a computer or play video games per day?**
a) 1 hour or less
b) 2 to 4 hours
c) More than 4 hours
d) Don't know

3. **How many hours do you sleep at night?**
a) less than 6 hours
b) 6 to 8 hours
c) 9 to 11 hours
d) I don't know

4. **Do you think it is important to eat a healthy diet?**
a) Yes
b) Yes, sometimes
c) No
d) I don't know

5. **Do you think it is important to be physically active?**
a) Yes
b) Yes, sometimes
c) No
d) I don't know

6. **Do you think that proper nutrition and physical activity are related to health?**
a) Yes
b) Yes, sometimes
c) No
d) I don't know

7. **How often do you eat your first meal (breakfast) at home?**
a) Every morning
b) 2-3 times a week
c) I don't eat before school
d) I don't know

8. **How many glasses of water do you drink per day?**
a) I don't drink water
b) 1-2 glasses
c) 3-4 glasses
d) 5 or more glasses

9. **How many servings of fruit do you eat per day?**
a) I didn't eat vegetables yesterday
b) Yes, I ate vegetables 1 time yesterday
c) Yes, I ate vegetables 2 times yesterday
d) Yes, I ate vegetables 3 or more times yesterday

10. **How often do you eat vegetables?**
a) I didn't eat vegetables yesterday
b) Yes, I ate vegetables 1 time yesterday
c) Yes, I ate vegetables 2 times yesterday
d) Yes, I ate vegetables 3 or more times yesterday

11. **Do you participate in any sport or physical activity?**
a) At least 60 minutes every day
b) 2-3 times a week for 60 minutes
c) I am rarely physically active
d) I don't know

Figure S1. Pilot-tested questionnaire for pupils in Grade 2, Grade 3, and Grade 4
