# Supplementary material for: Health-Related Behaviors and BMI Across Three School Years: Annual Bayesian Network Patterns During the PETICA Childhood Obesity Prevention Program
Source: Children (Basel). 2026 Sep 24;13(10):1298. doi: 10.3390/children13101298 (PMC13703612; doi:10.3390/children13101298)
Supplement: Supplementary file 1 [file children-13-01298-s001.zip › Table S1_ Frequencies of responses_PETICA.pdf]

Table S1. Frequencies of responses on habits and attitudes among pupils across Grades 2–4 (total sample and by sex), with tests of statistical significance.

Questions related to attitudes and habits	Frequency (%)												p-value	
	Girls				Boys				All					
Second graders (G2)	a	b	c	d	a	b	c	d	a	b	c	d	Girls vs Boys	all pupils
How much time do you spend watching TV per day?	65.0	9.8	0.9	24.3	58.1	15.7	7.2	19.0	61.5	12.8	4.1	21.6	0.0183	
How often do you play on a computer or play video games per day?	71.5	5.5	2.2	20.8	59.1	18.7	9.0	13.2	65.1	12.4	5.7	16.8	0.0001	
How many hours do you sleep at night?	2.6	20.0	57.5	19.8	4.8	27.9	51.1	16.2	3.7	24.0	54.3	18.0	0.1834	
Do you think it is important to eat a healthy diet?	98.2	1.5	0.2	0.0	97.8	1.3	0.4	0.0	98.0	1.4	0.3	0.2	0.9819	
Do you think it is important to be physically active?	97.4	2.0	0.4	0.2	96.8	2.4	0.9	0.0	97.1	2.2	0.7	0.1	0.2660	
Do you think that proper nutrition and physical activity are related to health?	97.6	0.7	0.7	1.1	94.8	0.6	2.2	2.4	96.2	0.7	1.4	1.7	0.6092	
How often do you eat your first meal (breakfast) at home?	78.4	21.5	0.0	0.0	71.6	28.3	0.0	0.0	75.0	25.0	0.0	0.0	0.5164	
How many glasses of water do you drink per day?	0.0	6.1	16.7	77.2	0.4	8.0	18.4	73.2	0.2	7.1	17.5	75.2	0.7466	
How many servings of fruit do you eat per day?	1.8	13.8	77.0	7.5	3.7	17.9	67.9	10.5	2.7	15.9	72.4	9.0	0.2624	
How often do you eat vegetables?	2.7	30.1	54.4	12.8	6.0	35.0	44.1	14.9	4.4	32.6	49.2	13.9	0.1575	
Do you participate in any sport or physical activity?	43.3	42.0	3.8	10.9	43.4	40.8	7.0	8.8	43.4	41.4	5.4	9.8	0.5725	
Third graders (G3)														G2 vs G3
How much time do you spend watching TV per day?	63.9	17.3	2.0	16.1	57.4	23.4	5.9	12.2	61.2	20.3	3.9	14.2	0.1045	0.0843
How often do you play on a computer or play video games per day?	86.1	5.8	0.9	6.7	54.3	27.3	8.1	9.7	70.3	16.5	4.5	8.2	<0.0001	0.0132
How many hours do you sleep at night?	1.1	21.1	65.5	11.7	2.5	24.5	61.0	11.5	1.8	22.8	63.3	11.6	0.6614	0.0758
Do you think it is important to eat a healthy diet?	98.9	0.9	0.2	0.0	96.6	3.2	0.2	0.0	97.8	2.0	0.2	0.0	0.6352	0.5976
Do you think it is important to be physically active?	96.9	3.1	0.0	0.0	96.2	3.8	0.0	0.0	96.5	3.5	0.0	0.0	0.9875	<0.0001

Do you think that proper nutrition and physical activity are related to health?	98.4	1.1	0.2	0.1	95.7	2.3	2.0	0.0	97.1	1.7	1.1	0.1	0.0070	<0.0001	
How often do you eat your first meal (breakfast) at home?	97.8	2.2	0.0	0.0	94.8	4.2	0.0	0.0	96.3	3.6	0.1	0.1	0.7898	<0.0001	
How many glasses of water do you drink per day?	0.2	4.7	21.3	73.3	0.5	5.9	22.7	69.8	0.3	5.3	22.0	71.6	0.8765	0.6267	
How many servings of fruit do you eat per day?	0.1	24.2	61.2	13.5	0.1	24.3	53.4	20.7	0.1	24.3	57.3	17.1	0.3025	<0.0001	
How often do you eat vegetables?	0.1	33.6	48.5	17.3	0.1	34.9	44.0	20.3	0.1	34.3	46.3	18.8	0.8129	<0.0001	
Do you participate in any sport or physical activity?	37.9	52.2	4.9	3.6	41.4	48.6	3.6	4.1	29.7	50.4	4.3	3.8	0.7788	0.0005	
Fourth graders (G4)														G2 vs G4	G3 vs G4
How much time do you spend watching TV per day?	61.6	17.5	2.2	17.3	52.9	24.1	4.4	17.5	57.2	20.8	3.3	17.4	0.2270	0.2029	0.8038
How often do you play on a computer or play video games per day?	79.9	9.5	1.9	8.1	50.4	33.8	8.0	6.1	64.2	21.7	5.0	7.1	<0.0001	0.0006	0.5629
How many hours do you sleep at night?	0.8	27.6	59.1	9.5	2.2	31.3	57.1	8.0	1.5	29.4	58.1	8.8	0.6415	0.0028	0.4076
Do you think it is important to eat a healthy diet?	99.2	0.8	0.0	0.0	97.0	2.2	0.1	0.1	98.1	1.8	0.1	0.1	0.7682	0.6173	0.9445
Do you think it is important to be physically active?	98.3	1.9	0.0	0.0	97.0	2.8	0.1	0.1	97.2	2.8	0.0	0.0	0.9183	<0.0001	0.9807
Do you think that proper nutrition and physical activity are related to health?	99.7	0.3	0.0	0.0	97.1	2.9	0.0	0.0	98.5	1.5	0.0	0.0	0.4935	<0.0001	<0.0001
How often do you eat your first meal (breakfast) at home?	92.5	7.5	0.0	0.0	90.9	9.1	0.0	0.0	91.7	8.3	0.0	0.0	0.9582	<0.0001	0.0528
How many glasses of water do you drink per day?	0.3	5.8	23.4	69.4	0.6	4.7	26.0	67.9	0.4	5.3	24.7	68.6	0.8731	0.3280	0.9294
How many servings of fruit do you eat per day?	11.4	26.2	61.0	0.4	18.3	25.5	55.1	1.1	14.9	25.8	58.0	1.3	0.2960	<0.0001	<0.0001
How often do you eat vegetables?	15.0	29.0	55.4	0.6	20.8	33.8	45.2	0.2	17.9	31.4	50.3	0.4	0.1447	<0.0001	<0.0001
Do you participate in any sport or physical activity?	37.9	54.0	2.8	3.3	41.8	49.3	3.9	3.0	39.9	51.7	3.3	3.2	0.7645	0.0006	0.3831
